# Application of Next Generation Sequencing (NGS) in Phage Displayed Peptide Selection to Support the Identification of Arsenic-Binding Motifs

**DOI:** 10.3390/v12121360

**Published:** 2020-11-27

**Authors:** Robert Braun, Nora Schönberger, Svenja Vinke, Franziska Lederer, Jörn Kalinowski, Katrin Pollmann

**Affiliations:** 1 Department of Biotechnology, Helmholtz Institute Freiberg for Resource Technology, Helmholtz Center Dresden-Rossendorf, 01328 Dresden, Germany; n.schoenberger@hzdr.de (N.S.); f.lederer@hzdr.de (F.L.); k.pollmann@hzdr.de (K.P.); 2 Microbial Genomics and Biotechnology, CeBiTec–Center for Biotechnology, Bielefeld University, 33594 Bielefeld, Germany; svenja.vinke@uni-bielefeld.de (S.V.); joern@CeBiTec.Uni-Bielefeld.DE (J.K.)

**Keywords:** phage display, peptide, biopanning, target-unrelated peptide, arsenic, motif, NGS, Illumina, interaction, oxyanion

## Abstract

Next generation sequencing (NGS) in combination with phage surface display (PSD) are powerful tools in the newly equipped molecular biology toolbox for the identification of specific target binding biomolecules. Application of PSD led to the discovery of manifold ligands in clinical and material research. However, limitations of traditional phage display hinder the identification process. Growth-based library biases and target-unrelated peptides often result in the dominance of parasitic sequences and the collapse of library diversity. This study describes the effective enrichment of specific peptide motifs potentially binding to arsenic as proof-of-concept using the combination of PSD and NGS. Arsenic is an environmental toxin, which is applied in various semiconductors as gallium arsenide and selective recovery of this element is crucial for recycling and remediation. The development of biomolecules as specific arsenic-binding sorbents is a new approach for its recovery. Usage of NGS for all biopanning fractions allowed for evaluation of motif enrichment, in-depth insight into the selection process and the discrimination of biopanning artefacts, e.g., the amplification-induced library-wide reduction in hydrophobic amino acid proportion. Application of bioinformatics tools led to the identification of an SxHS and a carboxy-terminal QxQ motif, which are potentially involved in the binding of arsenic. To the best of our knowledge, this is the first report of PSD combined with NGS of all relevant biopanning fractions.

## 1. Introduction

Arsenic is a toxic metalloid often used in semiconductor elements as gallium arsenide (GaAs) compound. It naturally occurs as a trace element at average concentrations of ~5 ppm, but is concentrated as part of many minerals. Anthropogenic or natural processes lead to the release and contamination of naturally occurring water bodies [1]. Human population in many countries are exposed to high levels of arsenic from water, including Taiwan, Argentina, Chile, Mexico, India, Bangladesh and Chile [2]. For many years now, the United States Agency for Toxic Substances and Disease Registry (ATSDR) classifies arsenic as most important, No. 1 ranked priority hazardous substance (https://www.atsdr.cdc.gov/SPL/index.html, 2020/08/05). Its main toxicity results from inorganic arsenate (HAsO_4_^2−^) mimicking phosphate (HPO_4_^2−^) and thus competition for and inhibition of phosphate transporters and phosphate-metabolizing enzymes, including essential metabolic processes like the oxidative phosphorylation to regenerate adenosine-5′-triphosphate (ATP) [3]. Exposure to arsenic also results in increased prevalence for lung, bladder and skin cancer [4]. However, recent industrial usage of arsenic in gallium arsenide and its increasing importance for the electronic industry in the production of LED’s and photovoltaics led to an increasing demand [5]. Efficient recovery and detection systems are both needed to meet the growing demand and to monitor and reduce toxic contaminations. Biological arsenic binding molecules may be used in biosensors and in future recycling systems.

In recent years, the application of phage display has led to the discovery of many peptide structures, with targets ranging from inorganics and solids to carcinoma cells [6]. However, although successfully applied in the identification of many target-binding molecules, phage display is prone to errors and notoriously known for the identification of false positive hits [7]. The unique power of phage display lies in the possibility for fast and efficient identification of ligands with affinity to a desired target material out of large populations of phage clones displaying billions of different randomized peptides on their surface. However, often target-unrelated peptide (TUP) sequences rather than specific binding sequences are identified. These sequences occur for many reasons, phage can bind to components of the laboratory experimental setup, e.g. to blocking or capture reagents [8]. Furthermore, the amplification of phage libraries between different rounds of library enrichment in bacteria is an essential step in the selection of ligands. However, it leads to the identification of recurrent phage clones with a propagation advantage rather than the selection of target-binding phage. Amplification also decreases the diversity of the library and it can strongly affect the identification of useful ligands. Thus, the distinction of identified ligands for either target-related or growth advantage-related selection pressure is a challenging, yet necessary obstacle in the implementation of phage display experiments. Traditional Sanger sequencing of a limited number of single clones leads to a loss of information and limits the ability to identify true positive target-binding ligands [7,9,10,11,12].

In this study, we used next generation sequencing (NGS) to gain in-depth insight into the various fractions of three rounds of biopanning against immobilized arsenic and to evaluate the target-specific and growth-advantage related selection pressure. We were able to identify amino acids and motifs frequently occurring in fast-propagating ligands, amino acids detrimental for growth and thus leading to reduced libraries and motifs, which are potentially binding to arsenic. Using bioinformatics tools and statistics, we could confirm position-specific amino acid patterns and compared the identified motifs to known structures to prevent identification of known target-unrelated peptides. Comparing traditional Sanger sequencing to the applied NGS, we could show the increased information content gained through extensive sequencing, ultimately leading to the discovery of novel potential arsenic-binding ligands. This study may help in the planning of future phage display experiments and in the implementation of NGS and bioinformatics tools to identify specific target-binding ligands.

## 2. Materials and Methods

### 2.1. Media and Buffer

In this work, the following media and buffer were used. Media: LB medium (10 g L^−1^ tryptone, 5 g L^−1^ yeast extract, 5 g L^−1^ sodium chloride), top agarose (LB medium containing 7 g L^−1^ agarose), IPTG-(Isopropyl-β-D-thiogalactoside)-Xgal-(5-Bromo-4-chloro-3-indolyl-β-D-galactoside) agar (LB medium containing 15 g L^−1^ agar, 0.05 g L^−1^ IPTG, 0.04 g L Xgal). Buffer: TBS (TRIS buffered saline solution, 50 mM Tris(hydroxymethyl)aminomethane hydrochloride, 150 mM sodium chloride, pH 7.5), PEG/NaCl solution (20% *w*/*v* Polyethylene glycol 8000, 2.5 M sodium chloride), NaOH/NaCl solution (1 M NaOH, 1 M NaCl), McIlvaine buffer [13] (230.25 mM disodium phosphate dihydrate, 7.9 mM citric acid, pH 7.5), BW (Binding&Wash) buffer 2x (10 mM TRIS HCl, 1 mM ETDA, 2 M NaCl pH 7.5).

### 2.2. Phage Library

The commercially available Ph.D.^TM^–12 phage library LOT 0151606 (New England Biolabs Inc., Ipswich, MA, USA) was used for the biopanning experiments described in this work. It is a combinatorial library composed of random linear 12-mer peptides fused to the n-terminal part of pIII, the minor coat protein of M13 bacteriophage. Please see the manufacturer’s product information for further details.

*Escherichia coli* K12 ER2738 (Genotyp F’*proA^+^B^+^ lacI^q^* Δ*(lacZ)M15 zzf::Tn*10(Tet^R^)*/fhuA2 glnV* *Δ(lac-proAB) thi-1 Δ(hsdS-mcrB)5*) was used for phage amplification and determination of numbers of infectious phage (titration). Titration and amplification of phage were performed as described by Schönberger et al., 2019. The main steps of chromatopanning and subsequent sequencing are described below, for detailed descriptions please refer to Schönberger et al., 2019 [14].

### 2.3. Biopanning

#### 2.3.1. Experimental Setup

The chromatopanning called biopanning procedure described here was modified from Schönberger et al., 2019 [14] and first published by Nian et al., 2010 [15]. Target material were arsenic oxyanions, arsenous acid and arsenous anions (H_3_AsO_3_^−^, H_2_AsO_4_^−^, HAsO_4_^2−^, AsO_4_^3−^) of trivalent As(III) and pentavalent As(V) immobilized on a monolithic ion exchange column (CIM^®^ QA Disk Monolithic Column, BIA Separations, Ajdovščina, Slovenia) in a chromatographic setup using an Äkta avant 25 FPLC system (GE Healthcare, Amersham, UK).

In this study, phage were incubated with the unloaded column in a pre-screening (negative biopanning) for removal of unspecific binding phage followed by enrichment of binding phage in three rounds of positive chromatopanning against the immobilized target material.

#### 2.3.2. Column Handling and Target Immobilization

Column and system preparation included disinfection prior to all rounds of biopanning by sequential application of 60 column volume (cv) NaOH/NaCl solution, 20 cv ultrapure water (Milli-Q^®^ Direct, Merck KGaA, Gernsheim, Germany), 20 cv isopropyl alcohol (30% (*v*/*v*) 2-propanol) and 20 cv ultrapure water at a flow rate (*Q*) of 1.5 cv min^−1^.

Column equilibration preceding target immobilization achieving optimal target binding conditions was performed with 40 cv McIlvaine buffer pH 7.5 [13]. Arsenic immobilization took place by cyclic application of 1000 µL 50 µM sodium arsenite (NaAsO_2_) for 20 cv. Removal of excess arsenite was achieved by washing the column with 40 cv McIlvaine buffer pH 7.5.

#### 2.3.3. Phage Library Application and Enrichment

Pre-screening: Prior to target-specific phage enrichment, a pre-screening (negative biopanning) against an unloaded column was conducted. After equilibration of column for 60 cv McIlvaine buffer pH 7.5 at *Q* = 3 cv min^−1^, cyclic application of 10 µL of original Ph.D.–12 library in 490 µL McIlvaine buffer pH 7.5 for 20 cv at *Q* = 1.5 cv min^−1^ was performed. Unbound and/or weakly bound phage were collected with McIlvaine buffer pH 7.5 (40 cv, *Q =* 3 cv min^−1^) and fractioned in 2 mL fractions. Phage titer of all fractions was determined. Phage-containing fractions were concentrated with Amicon^®^ Ultra-15 centrifugal filters (Merck KGaA, Darmstadt, Germany), amplified and used in the following biopanning round against immobilized arsenic. Remaining phage were removed with 1 M phosphoric acid (100 cv, *Q =* 3 cv min^−1^) and discarded prior the column disinfection.

Positive biopanning: Three rounds of biopanning against on-column immobilized arsenic were performed. The chromatographic run of each round included target immobilization followed by cyclic application of phage (20 cv, *Q =* 1.5 cv min^−1^), column wash for removal of weakly/non-binding phage (McIlvaine buffer pH 7.5, 40 cv, *Q =* 3 cv min^−1^), phage elution (2 M magnesium sulfate, 40 cv, *Q =* 3 cv min^−1^) and phage stripping (1 M phosphoric acid, 40 cv, *Q =* 3 cv min^−1^), arsenic removal (1 M hydrochloric acid, 20 cv, *Q =* 3 cv min^−1^) and column disinfection. Wash, elution and stripping steps were fractioned in 2 mL fractions. Phage titer of all fractions were determined. After each of the first two positive biopanning rounds, five phage-containing fractions of both, elution and stripping, were concentrated with Amicon^®^ Ultra-15 centrifugal filters (Merck KGaA, Darmstadt, Germany). The concentrate of stripping fractions was neutralized with 1 M Tris(hydroxymethyl)aminomethane hydrochloride (TRIS-HCl) pH 9.1. Concentrates were incubated with 300 µL freshly grown *Escherichia coli* K12 ER2738 (OD_600_~0.5) before phage amplification. Amplification times were 4.5 h after biopanning round 1 for concentrates of elution and stripping, 4.5 h for concentrate of elution and 18 h for the concentrate of stripping after biopanning round 2. The lengthened amplification of phage from the stripping concentrate required storage of the amplified phage from elution concentrate in 50% glycerol (*v*/*v*) after biopanning round 2.

Volumes of phage solution for on-column interaction with the target material were: 700 µL for biopanning round 1 (350 µL amplified phage in 350 µL McIlvaine buffer pH 7.5), 600 µL for biopanning round 2 (composed of 150 µL of amplified phage of elution and stripping concentrates, respectively, in 300 µL McIlvaine buffer pH 7.5) and 2400 µL for biopanning round 3 (composed of 500 µL amplified phage of elution concentrate, 100 µL of amplified phage of stripping concentrate, 1800 µL McIlvaine buffer pH 7.5).

The volume of the washing step for the removal of weakly/non-binding phage was increased to 80 cv in biopanning round 2, and 100 cv in biopanning round 3.

### 2.4. Sanger Sequencing

The identification of the displayed combinatorial peptide sequences of individual phage required the isolation of single clones. The detailed procedure and the oligonucleotide primers are described by Schönberger et al., 2019 [14]. Sanger sequencing was performed by GATC Biotech AG, Eurofins Genomics, Germany.

### 2.5. Illumina Sequencing

Next generation sequencing on instrument HiSeq 1500 (Illumina, San Diego, CA, USA) was performed using the manufacturer’s kit HiSeq Rapid SBS Kit v2 (FC-402-4022). Samples were prepared using the following oligonucleotides. RBS1-Seqfwd1_btnl: (Bio)-5′-AC ACG ACG CTC TTC CGA TCT NNN NGT TTC GGC CGA ACC TCC AC-3′, RBS2-Seqfwd2_btnl: (Bio) -5′-AC ACG ACG CTC TTC CGA TCT NNN NNG TTT CGG CCG AAC CTC CAC-3′, RBS3-Seqfwd3_btnl: (Bio)-5′-AC ACG ACG CTC TTC CGA TCT NNN NNN GTT TCG GCC GAA CCT CCA C-3′, RBS4-Seqfwd4_btnl: (Bio) -5′-AC ACG ACG CTC TTC CGA TCT NNN NNN NGT TTC GGC CGA ACC TCC AC-3′, RBS5-Seqrev1: 5′-CAG ACG TGT GCT CTT CCG ATC TNN NNG CTG AGG GTG ACG ATC CC-3′, RBS6-Seqrev2: 5′-CAG ACG TGT GCT CTT CCG ATC TNN NNN GCT GAG GGT GAC GAT CCC-3′, RBS7-Seqrev3: 5′-CAG ACG TGT GCT CTT CCG ATC TNN NNN NGC TGA GGG TGA CGA TCC C-3′, RBS8-Seqrev4: 5′-CAG ACG TGT GCT CTT CCG ATC TNN NNN NNG CTG AGG GTG ACG ATC CC-3′. Biotinylated primers (Bio) were used for subsequent purification with Streptavidin-labelled beads. Furthermore, the primers contained 4 to 8 N-positions to shift the fluorescence signal of similar nucleobases, enabling sequencing of samples with high identity.

Samples were PCR amplified with Q5 high fidelity polymerase (New England Biolabs Inc., Ipswich, MA, USA). Reaction mixtures were prepared according to manufacturer’s instructions. PCR conditions were: initial denaturation 30 s × 98 °C, 35 cycles of denaturation 10 s × 98 °C, annealing 30 s × 60 °C, elongation 30 s × 72 °C, followed by final elongation 120 s × 72 °C.

PCR products were purified using Dynabeads™ M-280 Streptavidin (Invitrogen™, Thermo Fisher Scientific Inc., Waltham, MA, USA). Dynabeads™ were resuspended in 5 µL 2x BW buffer to a final concentration of 5 µg µL^−1^. An equal volume of 5 µL biotinylated PCR product in distilled water was added. Samples were incubated for 15 min at room temperature under gentle rotation. Biotinylated DNA coated Dynabeads™ were separated with a magnet for min. 3 min and washed 3 times with 1x BW buffer. Washed DNA-coated Dynabeads™ were resuspended in elution buffer. Then, 1 µL of beads was used as template for KAPA Hifi PCR (F.Hoffmann-La Roche AG, Basel, Switzerland) to anneal NEBNext^®^ (New England Biolab Inc., San Diego, CA, USA) Illumina indices. PCR composition was 2x KAPA Hifi HotStart ReadyMix 25 µL, NEBNext^®^ multiplex primer (E6-F8) 5 µL, template bead coated with DNA 1 µL, nuclease-free water 19 µL). PCR conditions were: initial denaturation 180 s × 95 °C, 35 cycles of denaturation 20 s × 95 °C, annealing 30 s × 60 °C, elongation 60 s × 72 °C followed by final extension 60 s × 72 °C. Successful amplification was checked on an agarose gel (1% *w*/*v*) by gel electrophoresis. Correct sized fragments were purified with NEB Monarch^®^ gel extraction kit (New England Biolabs, Ipswich, MA, USA). Concentration of amplicons was determined using Qubit dsDNA assay (Thermo Fisher Scientific Inc., Waltham, MA, USA). Amplicons were pooled in equimolar concentrations and purified again from agarose gel (1% *w*/*v*) before sequencing using Illumina HiSeq2500 (Illumina, San Diego, CA, USA) in 2 × 300 bp multiplex configuration by paired read deep sequencing.

### 2.6. Bioinformatic Processing

#### 2.6.1. Analysis of Illumina Data

Geneious Prime^®^ 2020.1.1 (Biomatters, Auckland, New Zealand) was used for data processing after separation of FASTQ files for the barcode sequences, corresponding to the individual experiments. Alignment of F and R sequencing files, and merging of paired-ends was performed prior to quality trimming to Phred scores >30, which was performed with BBDuk. Sequences that included 5′-TCT CAC TCT–(XXX)x12–GGT GGA GGT were extracted, trimmed to their 12mer insert, translated taking into consideration the amber stop codon readthrough and counted for their abundance. Phylogenetic trees were calculated with Geneious Prime^®^ using Jukes-Cantor model with Neighbor-joining algorithm. The underlying multiple alignments were calculated using Clustal Omega [16].

#### 2.6.2. Sequence Evaluation

Core and singleton sequences were calculated using Microsoft Excel scripts. PuLSE was used for calculation of amino acid frequencies [17]. Logo calculation based on the statistical significance of the individual residues in context to a background frequency was performed with pLogo [18]. Motif calculation comparing two sets of sequences for discovery of motif enrichment was performed using MEME [19].

## 3. Results

### 3.1. Biopanning Experiments

Three biopanning rounds against arsenic oxyanions immobilized on quaternary amines were conducted in a chromatographic setup. In order to avoid unspecific binding to the chromatographic equipment and the column material, a preceding pre-screening (negative biopanning) was performed. Only the flowthrough of phage was applied for the positive biopanning against arsenic.

After the third round of biopanning, sequences of 34 single clones of both, elution and the stripping fraction were identified. Forty-six unique sequences were found, of which 43 sequences occurred once. In the following Table 1 sequences, isoelectric point (pI, calculated using ProtParam [20]) and occurrence of all sequences, identified with Sanger sequencing are presented. Only three sequences were identified more than one time, the peptides FHMPLTDPGQVQ (pI 5.08) and SIHSVTKGRYPV (pI 9.99) were both identified with a frequency of 11/68. The peptide MKAHHSQLYPRH (pI 9.99) was identified with a frequency of 2/68.

### 3.2. Illumina (NGS) Sequencing

Within three rounds of positive biopanning, phages of 12 different fractions were collected and occurring peptide sequences were identified using Illumina sequencing. Additionally, to quantify amplification-induced selection biases, the naïve phage library Ph.D.TM–12 LOT 0151606 (New England Biolabs Inc., Ipswich, MA, USA) and amplicons of itself were sequenced.

In this study, within the 14 sequenced fractions, 521,981 evaluable reads were obtained. Reads and sequences for all fractions are shown in Table 2. Read numbers vary between 133,163 reads for the naïve library and 2373 reads for the elution fraction of the third biopanning round. Variations can be explained by library reduction due to selection pressure over the biopanning rounds and by sample preparation for Illumina sequencing. Phage isolation includes precipitation steps in 20% PEG-8000 and 2.5 M NaCl. Concentrations of these substances were reduced by repeated washing, but may still hinder the following DNA amplification by PCR. Samples showing low read numbers had to be diluted before PCR amplification.

### 3.3. NGS Fraction Composition

**Figure A1:**
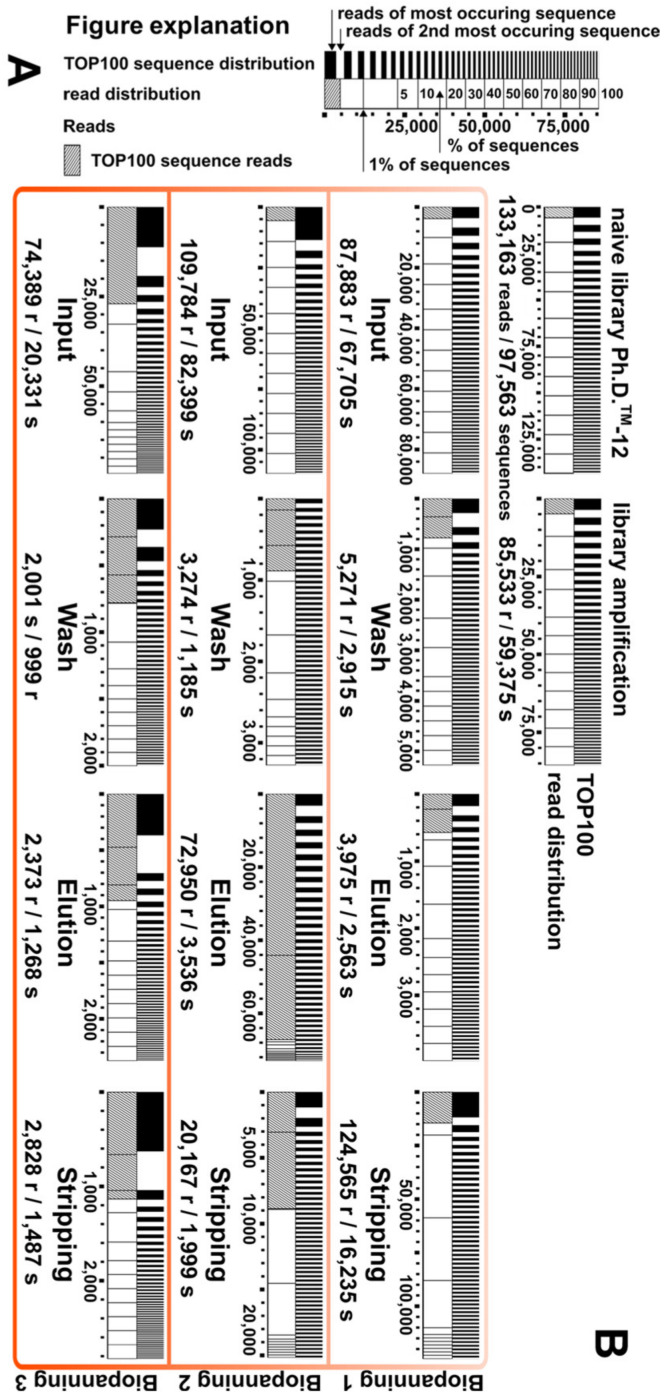
Read and sequence distribution of the fractions of three rounds of biopanning against on-column immobilized arsenic with Illumina sequencing (**B**). Top right (**A**), a figurative explanation is shown. The horizontal black stacked bar shows the distribution of the one hundred most occurring sequences relative to each other. Below in the white subdivided bar the read distribution is shown. Shown are 1%, 5%, 10%, 20%, …, 90%, 100% of the reads. The shaded area shows the read number occupied by the one hundred most occurring sequences. Read (r) and unique sequences (s) are given in numbers.

Due to the selection pressure, the composition of the fractions within the three rounds of biopanning changed. In Figure A1 in Appendix A, read and sequence distributions are shown for all fractions, that have been sequenced by Illumina sequencing. While the sequences are relatively equal distributed over the reads in the naïve library, the distribution is shifted towards a smaller number of more-abundant sequences dominating the reads of the fraction. Amplification of the fractions is subject to a different selection pressure, mainly from propagation rates and translation-based biases. Therefore, the shift is to some extent reversed after amplification (comparison of elution and stripping fractions to the input of the next biopanning round). The magnitude of distribution shift is larger in the first biopanning rounds, suggesting that the main target-binding selection takes place in these rounds. The distribution of reads within the one hundred most abundant sequences shifts towards the most-abundant sequences over the three rounds of biopanning.

### 3.4. Amino Acid Composition

**Figure 1:**
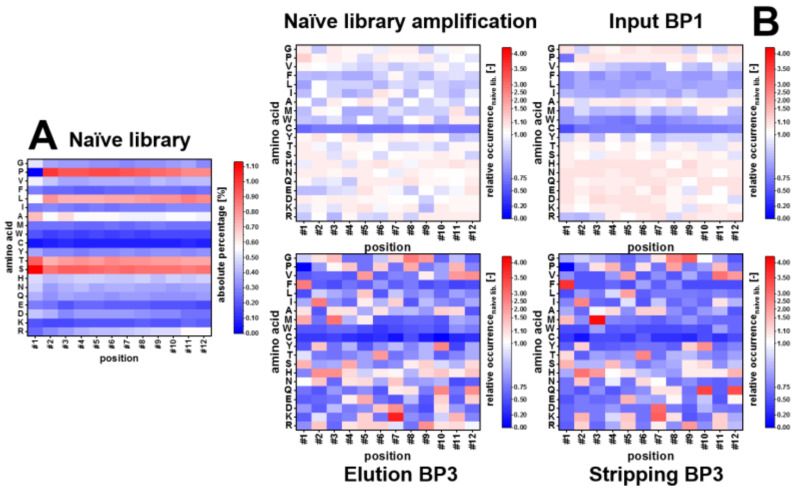
Comparison of the amino acid composition of selected fractions of three rounds of biopanning against on-column immobilized arsenic. Shown in heatmaps is the relative occurrence of each amino acid on each position of the randomized 12-mer peptide sequence, displayed on M13KE phage of the combinatorial Ph.D.^TM^–12 phage library (New England Biolabs, Ipswich, MA, USA) relative to the percentage of occurrence of the amino acids in the naïve library. The original amino acid percentage on each position of the naïve library is shown in (**A**). In (**B**) the relative occurrences of the following fractions are shown: amplification of the naïve library, input biopanning round 1 (BP1), elution and stripping biopanning round 3 (BP3). Figure A2, which shows heatmaps of all fractions can be found in Appendix A.

In order to quantify different selection pressures, which contribute to the library evolution over three cycles of biopanning, the determination of amino acid occurrence is necessary. In Figure 1 below, selected fractions are displayed in a heatmap showing the 20 amino acids and their respective occurrence in the relevant fraction (B). For comparison, the original amino acid percentage on each position of the randomized 12-mer sequence in the naïve library is shown (A). The occurrence of the individual amino acids in the naïve library corresponds to the manufacturer’s specifications with minor deviations (see www.neb.com for the amino acid frequency of Ph.D.^TM^ libraries). Most abundant amino acids are serine (10.80%), proline (10.06%), threonine (9.49%), leucine (9.13%) and alanine (6.87%). Least abundant amino acids are cysteine (0.99%), tryptophan (1.97%) and lysine (2.39%) (see complete table in Appendix A Table A1). It is noteworthy that the frequency of the individual amino acids is, in some cases, position-dependent. While, e.g., serine and alanine are found ~1.25-fold over its average frequency on position 1 (amino-terminal position of randomized 12-mer), arginine and lysine show a reduced frequency at position 1, which is steadily increasing to position 12. Lysine and proline show a highly reduced frequency at the n-terminal position (L 0.74-fold-, P 0.004-fold of average frequency).

The properties of amino acids mentioned in the following paragraph were adapted from Livingstone and Barton, 1993 [21]. The amplifications of the naïve library and after pre-panning (Input BP1) show, that polar amino acids threonine (T), serine (S), histidine (H), asparagine (N), glutamine (Q), glutamic acid (E), aspartic acid (D), lysine (K) and arginine (R) occur at most positions in high percentage compared to the naïve library. Occurrence of glutamic acid is reduced compared to the naïve library at the amino-terminal positions (1–4) of the linear 12-mer sequence, while arginine (R) shows the same frequency in the amplification after pre-panning.

Occurrence of cysteine is greatly reduced in all four fractions shown in Figure 1. Furthermore, the occurrence of many hydrophobic amino acids is reduced compared to the naïve library after amplification. Aliphatic, hydrophobic amino acids valine (V), leucine (L) and isoleucine (I) show a reduced occurrence after amplification compared to the amino acid frequencies in the naïve library. Hydrophobic aromatic amino acids phenylalanine (F), tryptophan (W) and tyrosine (Y) also show a reduced occurrence. Whereas the polar, hydrophobic amino acids threonine (T) and histidine (H) and the small, hydrophobic amino acids alanine (A) and glycine (G) show the same or higher frequencies as the naïve library after amplification, occurrence of hydrophobic methionine is reduced.

Within the three rounds of biopanning the relative frequencies of the amino acids become more fragmented; overarching trends for amino acid occurrences are reduced. Relative frequency of cysteine and tryptophan is highly reduced. Phenylalanine occurrence is reduced after the third biopanning round, though the relative frequency of the amino acid is strongly increased at the amino-terminal position 1. Aliphatic, hydrophobic amino acids valine, leucine and isoleucine generally show a reduced occurrence, with exceptions for valine (position 11), leucine (position 5) and isoleucine (position 2) after three rounds of biopanning. Further increased relative amino acid frequencies are threonine (position 6), serine (position 1, 4, 9), histidine (position 2, 3, 12), glutamine (position 10, 12), aspartic acid (position 7) and lysine (position 7).

### 3.5. Sequence Logo Calculation

**Figure 2:**
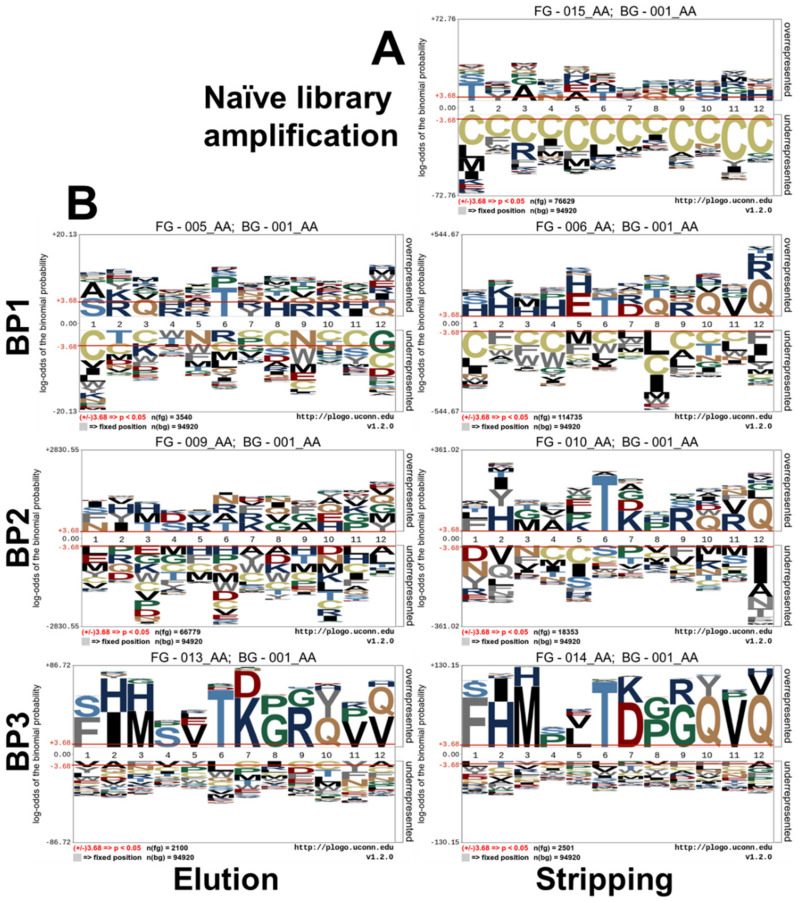
Sequence logos of selected fractions of three rounds of biopanning against on-column immobilized arsenic. Shown are logos, calculated using pLogo [18] based on the significance of the individual residues in context to the naïve phage library Ph.D.^TM^–12 as background frequency. (**A**) Amplification of the naïve phage library (**B**) Elution and stripping fractions of three rounds of biopanning showing the enrichment of the consensus sequence FHMPLTDPGQVQ.

**Figure A3:**
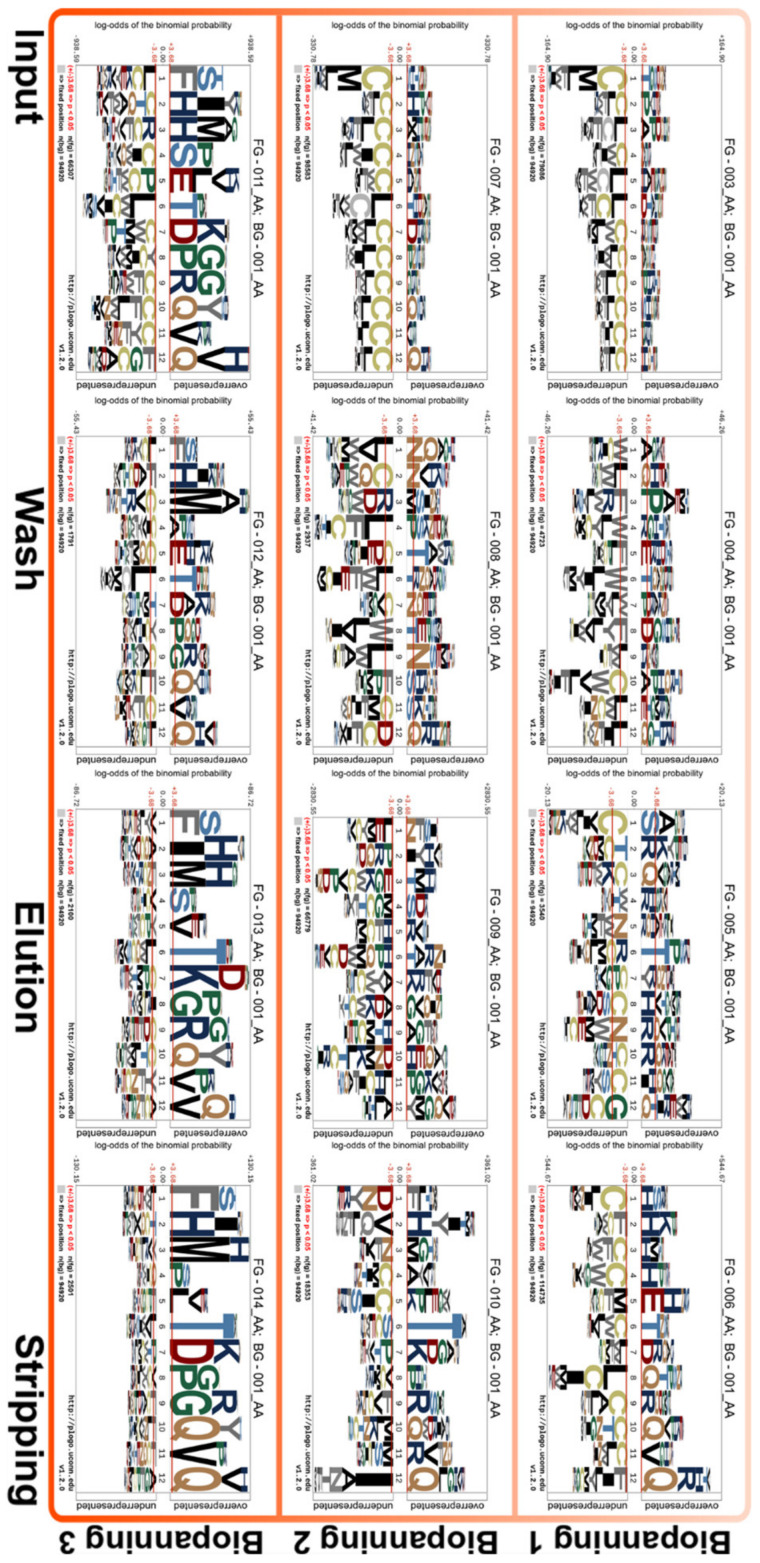
Logos of the fractions of three rounds of biopanning against on-column immobilized arsenic. Shown are logos, calculated using pLogo [18] based on the significance of the individual residues in context to the naïve phage library Ph.D.^TM^–12 as background frequency.

Calculation of amino acid occurrences based on the significance of the individual residues in context to the naïve phage library Ph.D.^TM^–12 as background frequency was performed with pLogo [18]. Special consideration was given to the elution and stripping fractions of each biopanning round to determine the efficiency of the biopanning rounds and to identify highly abundant amino acids and sequences. In the following Figure 2, logos generated with pLogo are shown for the amplification of the naïve library (A) and for elution and stripping fractions of biopanning round 1 and 3 (B). Logos generated for the fractions of all three biopanning round are shown in the Appendix A Figure A3.

The amplification of the naïve phage library shows that the amino acid frequency changes described in Section 3.4 occur at each position in the randomized 12-mer sequence. Whereas the relative frequency of cysteine (C) and the hydrophobic aromatic amino acids phenylalanine, tyrosine and tryptophan is reduced at all positions in the 12-mer, positive charged lysine and arginine are only reduced in the first n-terminal position (Position 1, 3). While occurrence of methionine is reduced in the first seven n-terminal positions, it shows an increased occurrence at position 11. Proline at position 1 is completely depleted after three rounds of biopanning, whereas its overall percentage over all positions within the fraction remains relatively unchanged (1.06× fold enrichment over naïve phage library). Cysteine is depleted at each position (0.36× fold of naïve phage library occurrence).

Elution and stripping fractions of BP1 and BP3 clearly indicate the emergence of the consensus sequence FxMPLTDGQVQ (with x being a hydrophobic amino acid) in the stripping fraction of the first biopanning round. This sequence is further enriched in the following biopanning rounds. Within the stripping of the first biopanning round, over representation of histidine in the amino-terminal part of the sequence is found.

### 3.6. Core Fraction Calculation

The evaluation of limited sets of sequences to quantify phage display experiments is always impeded by the large number of sequences covered in phage display libraries. A phage library displaying a random dodecapeptide allows for 4.1 × 10^15^ theoretically possible unique sequences. According to New England Biolabs, Ph.D.^TM^–12 libraries are delivered consisting of approximately 10^9^ unique sequences (covering ~2.5 × 10^−5^% of theoretically possible sequences) [22]. Even assuming that the library is equally distributed and that all sequences occur evenly, sequencing of 200,000 reads can only cover ~5 × 10^−9^% of all possible sequences. Phage display experiments sequencing 100 single clones therefore only cover ~2.5 × 10^−12^% of all possible sequences. Thus, a high level of selection pressure and enrichment must be assumed to allow for evaluation and identification of target-binding sequences. Enrichment follows amplification-induced selection and targeted selection of binding sequences. Provided a high level of selection pressure, sequences that are subject to the respective pressure, dominate the library. Each amplification step therefore advantages fast-propagating sequences, each elution and stripping step target-binding sequences (which may be fast-propagating). To harvest frequently occurring sequences, core sequences (intersections) including sequences found in all respective fractions were calculated.

Intersection of sequences from all three biopanning rounds were calculated to be:

(1) $$\cap \;E=E\left(BP1\right)\;\cap \;E\left(BP2\right)\;\cap \;E\left(BP3\right)$$

(2) $$\;\cap \;I=I\left(BP1\right)\;\cap \;I\left(BP2\right)\;\cap \;I\left(BP3\right)$$

(3) $$\;\cap \;W=W\left(BP1\right)\;\cap \;W\left(BP2\right)\;\cap \;W\left(BP3\right)$$

(4) $$\;\cap \;S=S\left(BP1\right)\;\cap \;S\left(BP2\right)\;\cap \;S\left(BP3\right)$$

The core sequences of the elution fraction (∩E) should include target-binding sequences, enriched through target-binding selection pressure. Contrary, core sequences of the input fractions (∩I) are subject to amplification-based selection pressure. The core sequences of wash fractions (∩W) include recurrent, low-binding and/or fast-propagating sequences. High frequent target-binding sequences may be found in this fraction, too. Stripping with phosphoric acid was carried out in order to elute strong target-binding sequences, which have not been eluted before. Thus, the core sequences of these fractions (∩S) include sequences with potential high-affinity binders.

For further reduction of eligible sequences, the following sets were calculated:

(5) ES= ∩ E ∪ ∩S

(6) ES\W=( ∩E ∪ ∩S )\∩W

(7) ES\I=( ∩E ∪ ∩S )\∩I

(8) ES − I − W=(( ∩E ∪ ∩S )\∩I )\∩W

(9) $$\mathrm{ES}\;-\;I\;-\;W\backslash \mathrm{na}ï.\mathrm{lib}._{\mathrm{TOP}10\%}=(((\;\cap E\;\cup \;\cap S\;)\backslash \cap I\;)\backslash \cap W\;)\backslash \mathrm{na}ï.\mathrm{lib}._{\mathrm{TOP}10\%}$$

(10) $$\mathrm{ES}\;-\;I\;-\;W\backslash \mathrm{na}ï.\mathrm{lib}._{\mathrm{TOP}25\%}=(((\;\cap E\;\cup \;\cap S\;)\backslash \cap I\;)\;\backslash \;\cap W\;)\backslash \mathrm{na}ï.\mathrm{lib}._{\mathrm{TOP}25\%}$$

The union ES of sets ∩E and ∩S was calculated to include all potentially target-binding sequences. Differences of ES and the sets ∩I, ∩W were calculated to remove fast-propagation, potentially non- or weak target-binding sequences. Removal of sequences with a natural selection advantage (because of high-frequent occurrence in the naïve phage library) was achieved by calculating the difference of the set ES–I–W and the most (10%, 25% of sequences) occurring sequences in the naïve phage library. In the following Table 3, read and unique sequence quantities of the calculated sets are given. Read numbers refer to the fraction, in which the sequence was first identified within the biopanning process (elution and stripping fraction of biopanning round 1 for set ES).

The core fractions differ strongly in their size. The input core fraction (∩I) contains 2912 sequences, compared to the elution core fraction (∩E) with 56 unique sequences. This difference in size can be partially explained with the size of the included original fractions of the phage display experiment. The size of the intersection of sets is limited by the smallest set (input BP1: 67,705 sequences, input BP2: 82,399 seq., input BP3: 20,331 sequences compared to elution BP1: 2563 sequences, elution BP2: 3536 sequences, elution BP3: 1268 sequences). Different read numbers of the intersection ES of ∩E and ∩S compared to the original core fractions are the result of unique sequences, included in both core fractions, which possess different read numbers in the respective fractions. The read numbers of core fraction ES presented in Table 3 refer to the read number of the elution fraction of the first biopanning round. By subtracting the input and wash core fractions, the number of unique sequences is further reduced. Indeed, by subtracting the input core fraction, no further reduction of the dataset occurs when subtracting the wash core fraction and/or the 10% of most occurring sequences in the naïve phage library, as these sequences are included in the input core fraction. Further reduction by subtraction of the top 25% most occurring sequences results in the loss of only one sequence.

**Figure 3:**
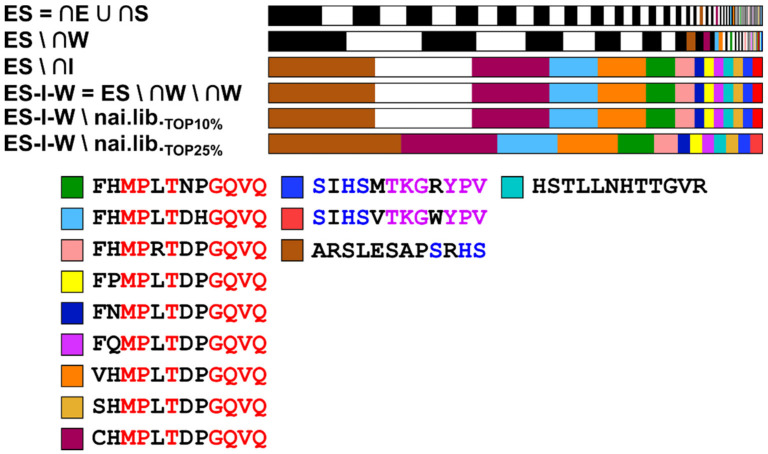
Visualization of the relative frequency of the unique sequences in the core fraction ES–I–W\naï.lib._TOP25%_ compared to the frequency of the respective sequences in the beforehand calculated core fractions. The horizontal stacked bars represent the total read number of each fraction, individual sequences are colored black/white and sorted from left to right proportional to their abundance. The size of the marked area is proportional to the frequency of the individual sequences. The area of specific sequences is colored. In total, 9/13 sequences of the core fraction ES–I–W\naï.lib._TOP25%_ carry the motif xxMPxTxxGQVQ (with x being any amino acid), 3/13 carry the motif SxHS either amino- or carboxy-terminal, 2/13 carry the motif SIHSxTKGxYPV, the remaining sequence does not show similarity to the other identified sequences and is rich in threonine, histidine and leucine. The enrichment process of the sequences shows that they are low abundant and become visible by subtraction of sequences with higher abundance.

The origin and the frequency of the remaining sequences can be visualized using stacked bars, which describe the composition of the respective fraction and the relative frequency of the unique sequences included in the fraction. In the following Figure 3, the calculated core fractions are shown. The fraction $\mathrm{ES}\;-\;I\;-\;W\backslash \mathrm{na}ï.\mathrm{lib}._{\mathrm{TOP}25\%}$ consists of 13 unique sequences. Nine of these sequences possess very similar amino acid motifs, all of them carry the consensus sequence xxMPxTxxGQVQ (with x being any amino acid). Furthermore, three of the remaining sequences carry the motif SxHS. The remaining sequence does not show similarities to the other identified sequences. It shows a high content of histidine, leucine and threonine. All thirteen sequences possess a small relative frequency in the core fraction ES and are continuously enriched by subtraction of the other core fractions, which remove the more abundant sequences.

### 3.7. Sequence Motif Calculation and Comparison

**Figure 4:**
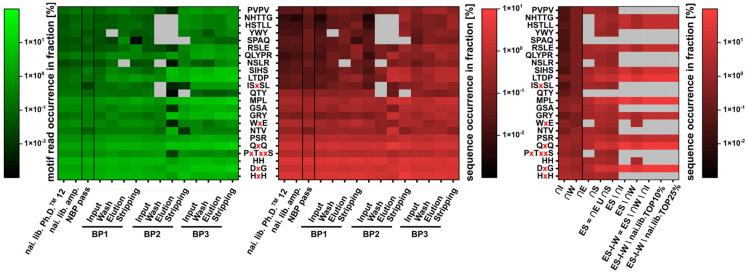
Sequence motif occurrence in reads (green), fractions of the three biopanning rounds (red, middle) and in the calculated core fractions (red, right). Motifs were calculated using MEME [19]. Shown are: the naïve Ph.D.^TM^–12 library, the amplification of the naïve library (naï. lib. amp.) and the pass of the preceding pre-panning (negative), which was used after amplification as input for the three rounds of biopanning against on-column immobilized arsenic. For the three biopanning rounds, the respective input, wash, elution and stripping fraction are shown as well as the core fractions calculated in Section 3.6.

The identification of motifs was performed using MEME [19]. A differential enrichment mode with a minimum width of 3 residues and 2 sites was chosen. Motifs with more than 5 sites or a width over 6 residues were ignored in the subsequent processing. The elution and stripping fractions of all three biopanning rounds were compared to the naïve phage library resulting in the identification of 22 motifs. Motif occurrence in unique sequences and reads was determined for all fractions. For the core fractions, motif occurrence was determined for the unique sequences. Read numbers were not determined for the core fractions, as the calculation of core fractions composed of more than one fraction results in read overlap for shared sequences. The motif occurrence is shown in the following Figure 4.

In the figure, the motif occurrence in reads is shown in green and the motif occurrence in the unique sequences is shown in red, with low to high abundance from dark to light. Grey areas show fractions, in which the motifs have not been identified. Most motifs, discovered with MEME, show an enrichment over the three rounds of biopanning. The motifs QTY and PxTxxS, however, were depleted in the biopanning process. Discovery over MEME might be due to overrepresentation of the motifs in individual fractions. The motifs HxH and HH, containing two adjacent histidines are enriched over the three biopanning rounds in reads and sequences. However, when calculating the core fractions, sequences containing HxH or HH were discarded in the subtraction of the input core fraction, indicating these sequences to be fast-propagating. Another motif lost in the subtraction of the input core fraction is PVPV, which was beforehand enriched in the biopanning procedure. The motifs MPL, LTDP, DxG and QxQ belong to the sequence family with the consensus sequence xxMPxTxxGQVQ. They are enriched in both the biopanning and the calculation of core sequences. The motif PxTxxS belongs to the family, too, but is depleted after subtraction of the input core fraction, indicating sequences carrying this motif to have a growth advantage. Motifs NHTTG and HSTLL belong to the sequence HSTLLNHTTGVR and are thus enriched. It is noteworthy that these sequences were found in all three stripping fractions but did not appear in any elution fractions. Motifs SIHS and GRY belong to the family of SxHSxTKGxYPV sequences, and RSLE to the sequence ARSLESAPSRHS.

**Figure 5:**
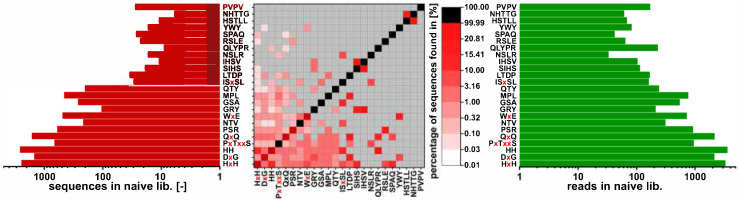
Visualization of the percentage of motif-bearing sequences, in which a second motif can be found. The population of sequences to be compared is defined in the X-axis, motifs which are compared for their appearance in the respective population in the Y-axis. In red bars the number of sequences, carrying the motif is given, in green bars the number of reads of the sequences carrying the motif is given. Motif comparisons colored dark red show that these occur multiple times in the sequences, leading to percentages of >100%. Calculations were performed with the sequence set of the naïve Ph.D.^TM^–12 library.

Sequences carrying the motif discovered with MEME were compared with each other to identify sequences with intersecting motifs. These sequences might be interesting for further characterization as they possess multiple structures that were enriched in the biopanning process. In Figure 5, all motifs are visualized for their appearance in sequences, which bear other motifs in the naïve Ph.D. ^TM^–12 library. For comparability, sequence and read numbers for the motif-carrying sequences are given. Calculation of the percentage of sequences in which a motif can be found in a population of low-frequent sequences show a smaller probability for intersecting motifs compared to high-frequent motif-bearing sequence populations. In the population of QxQ-bearing sequences, 29 sequences (1.78% of QxQ-bearing sequences) carrying an additional HxH motif (e.g., SQYDVNSSHQHQ) and 36 sequences (2.22% of QxQ-bearing sequences) carrying an additional HH motif (e.g., QTQFALHHLPSL) can be found. In comparison, in the population of ISxSL-carrying sequences, 1 sequence (3.33% of ISxSL-bearing sequences) shows an additional HxH motif (HHHHISHSLQLV), 2 sequences (6.66% of ISxSL-bearing sequences) possess an additional HH motif (IDSTKHHISRSL). Sequences, which possess combinations of motifs that were enriched in the biopanning process, might be considered when searching for candidate sequences with target-binding affinity. As the motifs SxHS and QxQ were prominent after three rounds of biopanning and further enriched through the calculation of the core fractions, sequences carrying both motifs might be of interest, even if they were lost in the biopanning process. Sequences, which were identified in the naïve library that carry both motifs, are QLQLDMDLSLHS and YQQQTSLHSPYA. However, both sequences were not found in any other fraction of the biopanning process. As the sequence QxQ was only found in a carboxy-terminal position of the randomized 12-mer peptide display in the phage library, the position of the motifs might be important to allow for peptide folding, necessary for binding.

### 3.8. QxQ Motif

Glutamine is not regularly described as a metal-binding amino acids, however in the sequences identified in this work, it was found in high frequency and in a defined position at the carboxy-terminal part of the randomized 12-mer, displayed by the phage library. The enrichment of sequences, carrying the motif xxxxxxxxxQxQ with two glutamines fixed on the positions 10 and 12 was determined.

**Figure 6:**
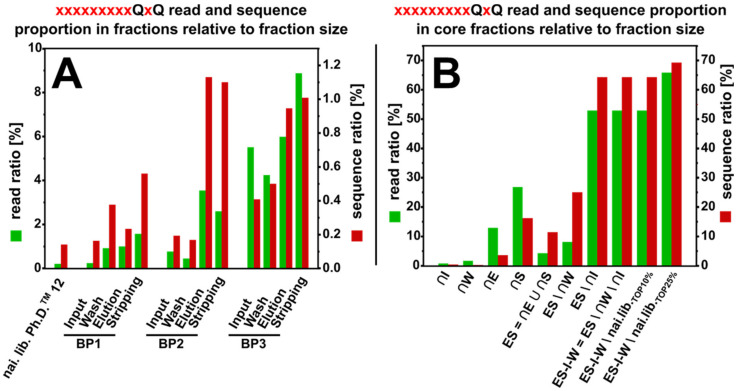
Occurrence of sequences carrying the motif xxxxxxxxxQxQ with two carboxy-terminal glutamines on positions 10 and 12 of the randomized 12-mer display on the Ph.D.^TM^–12 phage library. The occurrence in reads (green) and sequences (red) of the respective fraction of three rounds of biopanning against on-column immobilized arsenic (**A**) and of the calculated core fractions (**B**) is shown.

In Figure 6, the frequency of the motif-carrying sequences over the three rounds of biopanning (A) and in the calculated core fractions (B) is shown. Sequences, carrying the motif make up 0.21% of the reads of the naïve library sequencing and 0.14% of the sequences. After three rounds of biopanning, in which the motif occurrence is constantly increased, the motif-bearing sequences make up 5.98% of the reads and 0.95% of the sequences in the elution fraction, and 8.87% of the reads and 1.01% of the sequences in the stripping fraction. In the core fractions, occurrence in ∩E (12.92% of the reads, 3.57% of the sequences) and ∩S (26.85% of the reads, 16.22% of the sequences) is further increased, whereas the motif-carrying sequences show a smaller occurrence in ∩I (0.78% of the reads, 0.37% of the sequences) and ∩W ((1.70% of the reads, 0.26% of the sequences). In the final core fraction ES–I–W\naï.lib._TOP25%_, the occurrence is 65.85% of the reads and 69.23% of the sequences. As previously explained, read interpretation is difficult as the read numbers originate from the fraction, where the sequence was identified first (elution and stripping of first biopanning round for ∩E and ∩S).

Amplification-based selection advantage would result in the increased occurrence of xxxxxxxxxQxQ motif-carrying sequences after phage propagation. However, the occurrence at the beginning of each biopanning round after amplification is reduced compared to the elution and stripping fraction of the preceding biopanning, indicating a target-binding based enrichment. Furthermore, in each biopanning round, the motif-bearing sequences can be found primarily in the elution and stripping fraction, whereas the sequences are less frequent in the wash fraction, indicating that the selection is not based on weak, unspecific binding. It is noteworthy that the occurrence of motif-carrying sequences is slightly higher in the stripping fractions and especially in ∩S when compared to the corresponding elution fractions and ∩E. This might be the result of the respective eluent used in the process. Whereas 2 M magnesium sulfate was used for elution, phages were stripped from the column with 1 M phosphoric acid in the stripping fractions. The pH change of the stripping might result in a less efficient binding and promote the recovery of phage from the column.

**Figure 7:**
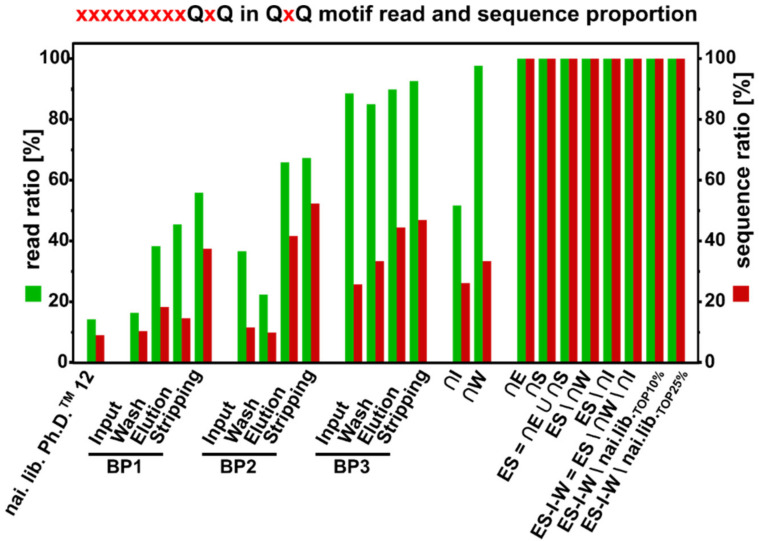
Proportion of reads (green) and sequences (red) carrying the motif xxxxxxxxxQxQ with two fixed carboxy-terminal glutamines relative to all reads and sequences carrying QxQ on random positions for three rounds of biopanning against on-column immobilized arsenic and of the calculated core fractions.

Unlike the SxHS motif, which was found both amino- and carboxy-terminal within the randomized 12-mer, 9/13 identified sequences in the final core fraction did possess two exclusively carboxy-terminal glutamine residues. Therefore, the frequency of the sequences containing the carboxy-terminal QxQ motif was calculated relative to QxQ-carrying sequences on random positions, shown in Figure 7. While covering 9.11% of all sequences and 14.26% of all reads of all QxQ-carrying sequences, the relative frequency of sequences with the carboxy-terminal QxQ increases over the three biopanning rounds. The highest relative frequency in the three biopanning rounds was found in the stripping fraction of the third round (92.62% of reads, 33.34% of sequences). Although the read and sequence ratio are being reduced in ∩I, in ∩W the numbers resemble the stripping fraction of the third biopanning round. In the core fractions ∩E and ∩S sequences carrying carboxy-terminal motifs cover 100% of reads and sequences of all QxQ carrying sequences. Amplification leads to a reduction in the relative frequency as seen in the amplification steps after the first and second biopanning round. Interestingly, the overall proportion of carboxy-terminal QxQ-motif carrying sequences in all QxQ-motif carrying sequences increases over all three biopanning rounds, as well as in the calculated core fractions. This might be an indication for the presence of other residues on fixed positions in the 12-mer which, together with the carboxy-terminal QxQ, form a structure that is beneficial for binding the immobilized oxyanions of arsenic. Other fixed positions in the consensus sequence xxPxTxxGQVQ may be necessary to allow the binding of the target material.

**Figure A4:**
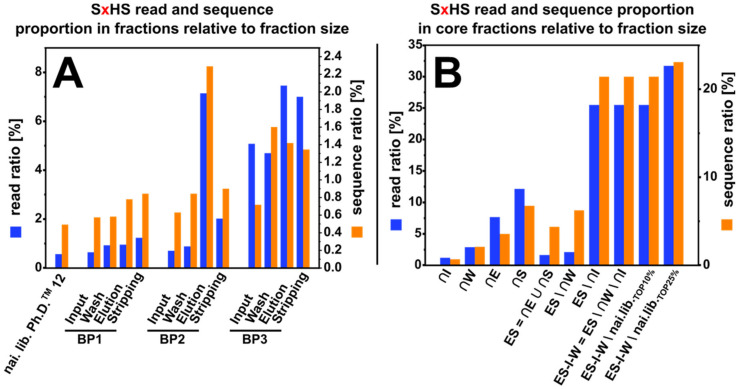
Occurrence of sequences carrying the motif SxHS in the randomized 12-mer displayed on the Ph.D.^TM^–12 phage library. The occurrence in reads (blue) and sequences (orange) of the respective fraction of three rounds of biopanning against on-column immobilized arsenic (**A**) and of the calculated core fractions (**B**) is shown.

**Figure A5:**
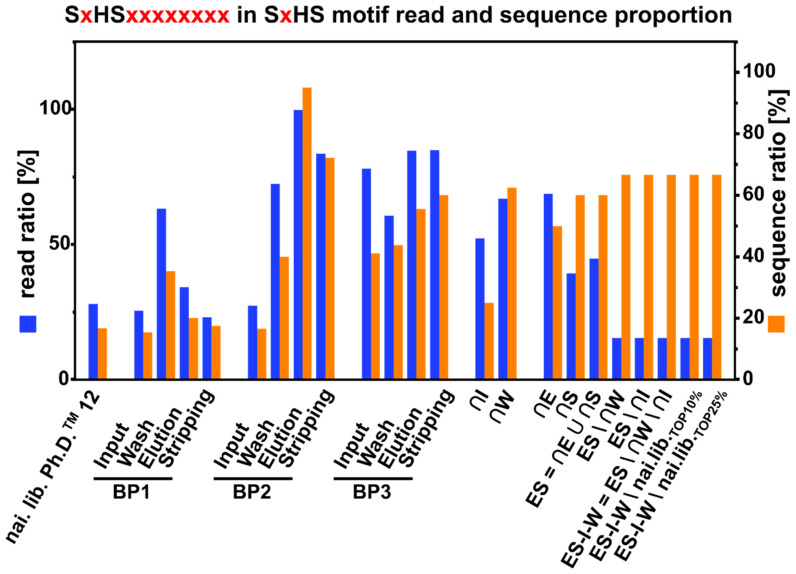
Proportion of reads (blue) and sequences (orange) carrying the motif SxHSxxxxxxxx relative to all reads and sequences carrying SxHS on random positions for three rounds of biopanning against on-column immobilized arsenic and of the calculated core fractions.

This comparison of motif occurrences and enrichment in different fractions and position-specificity was performed for SxHS motifs, too and can be found in Figure A4 and Figure A5 in Appendix A.

### 3.9. Motif Comparison with 48 h Discovery Database

**Figure A6:**
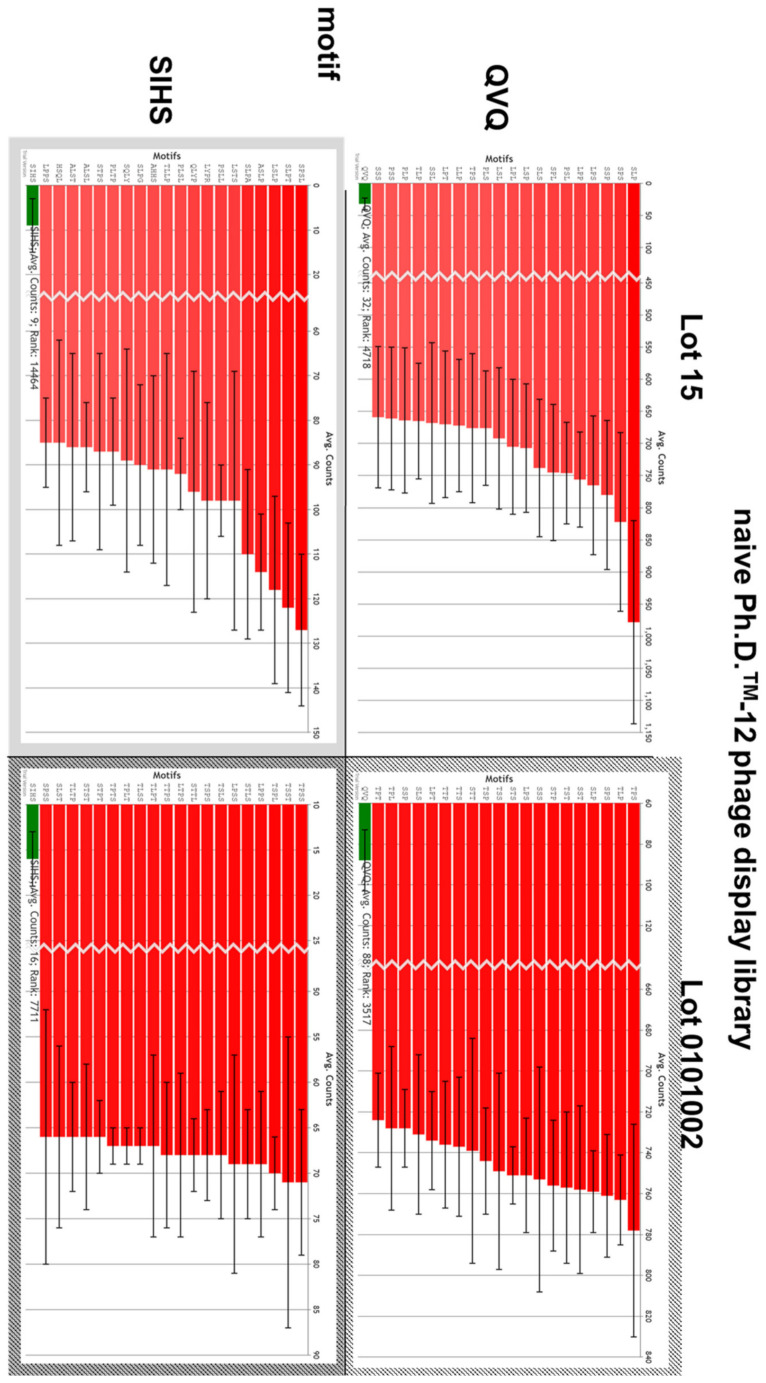
Comparison of motif occurrence in two different lots of the naïve Ph.D.^TM^–12 phage library (which were not used in this work) on 48hd.cloud [23]. Motifs QxQ and SxHS (green) are shown in comparison to the respective most abundant motifs (red).

Prof. Ratmir Derda (University of Alberta, CA, USA) granted us access to his recently published database www.48hd.cloud, which is still in development [23]. This database is currently the largest available repository for next generation sequencing results of phage surface display experiments. It allows setup of experiments, sample structuring and quantification of results with extensive statistical evaluation. It is also possible to perform motif analysis to determine motif frequencies within datasets. To evaluate the data obtained in this work, motif occurrence of QVQ and SIHS was compared to two naïve Ph.D.^TM^–12 library lots. A visualization of the data can be found in Appendix A, Figure A6. The ten most common, three residues long motifs in both lots of the library were SLP, SPS, TPS and TPL. The motif QVQ had a small read count of ~0.03x fold compared to the most common motif SLP in lot 15 and a ~0.10x fold read count compared to the most common motif TPS in lot 0101002, resulting in an oval rank within three residues long reads of 4718 in the first lot and rank 3517 in the second lot. As lot 15 was sequenced multiple times, the mean rank of QVQ in this library was 4557 ± 254. Motif SIHS ranked 16551 ± 2694 among 4r residues long motifs in lot 15 and rank 7717 in lot 0101002. These findings show that both motifs are not among the most abundant motifs in the naïve library, even when compared with other lots. Both motifs are enriched over the biopanning process and in the core fractions, indicating a directed selection of sequences carrying these motifs.

## 4. Discussion

### 4.1. Comparison of Sanger Sequencing with Next Generation Sequencing

Generally, phage display and biopanning results are influenced by different selection pressures in the phage accumulation. Propagation rates and translation-based biases (amplification-induced selection) compete with target-binding selection. Furthermore, unspecific and/or low-binding peptide sequences skew the identification of specific-binding sequences. These biases lead to reduced phage library diversity, decreasing library size and distorted library distribution [7,22,24].

Sequencing of a limited number of sequences occurring in phage libraries can only identify very small parts of the complete library. The linear combinatorial 12-mer library Ph.D.^TM^–12 theoretically possesses ~4 · 10^15^ individual sequences and is provided by the manufacturer comprising of ~10^9^ sequences. Even extensive Illumina sequencing can therefore cover only parts of the naïve library composition. A central question of this work was whether or not limited Illumina sequencing (<10^6^ reads) is able to provide additional information and thus enhance identification of potentially specific-binding sequences.

Three rounds of biopanning in a chromatographic setup were performed against on-column immobilized arsenic oxyanions (occurring mostly as As(V) H_2_AsO_4_^−^/HAsO_4_^2−^) [25]. The three most abundant sequences FHMPLTDPGQVQ (11/68), SIHSVTKGRYPV (11/68) and MKAHHSQLYPRH (2/68) after Sanger sequencing of 68 single clones are high abundant sequences in the naïve library and its amplification, too. In Table 4, the frequencies of these three sequences are given for the Illumina sequencing results of the fractions: (1) naïve library, (2) amplification of naïve library, (3) input into the three rounds of biopanning, (4) elution fraction of third biopanning round, (5) stripping fraction of third biopanning round in comparison to the results of Sanger sequencing (0). All three sequences are enriched over the three rounds of biopanning; however, FHMPLTDPGQVQ (~89x fold enrichment) and SIHSVTKGRYPV (~77x fold) show higher abundance in the elution and stripping fractions of the third biopanning round compared to MKAHHSQLYPRH (~10x fold). Increasing occurrence of these sequences may be partially explained by smaller read numbers of the third biopanning fractions (compare Table 2). The lower the read number of a fraction is, the higher the probability to identify high abundant sequences. However, enrichment of FHMPLTDPGQVQ and SIHSVTKGRYPV is stronger compared to MKAHHSQLYPRH, indicating a directed selection pressure, occurring because of either amplification-based selection advantages and/or target-binding selection advantages. Sequences may very well be fast-propagating and specific target-binding, resulting in a high selection pressure towards these sequences. Sequences with equally well binding properties to the target material but with growth advantage will always outcompete slower propagating sequences with the same binding properties [26]. Biological explanations for growth advantages include binding to the pili, usage of rare codons and motifs interfering with transport and infection. Libraries displaying peptides on the PIII protein are normally less affected by growth advantage-based biases compared to PVIII libraries [9,22,27].

Although subjected to different selection pressure, most often found sequences after Sanger sequencing have been high abundant in the original naïve library, too. Three rounds of biopanning changed the relative occurrence of high abundant sequences. However, no sequences have been identified more than once, that initially occur in low abundance. Careful consideration is needed in the selection of sequences for subsequent binding experiments, as high abundant sequences with high selection advantage may parasite the biopanning, leading to false-positive identifications.

Motifs, which were identified with Illumina sequencing, bioinformatics processing using tools as PuLSE [17], pLogo [18], MEME [19] and core fraction calculation for the further reduction of sequence sets, are identical to the motifs discovered using Sanger sequencing. These findings suggest that enrichment of binding sequences over three biopanning rounds was sufficient for the discovery of key motifs. However, comparison also clearly indicates the shortcomings of Sanger sequencing, only. Identification of sequence variations, key motifs and quantification of sequences, concerning the underlying selection pressure are not possible.

### 4.2. NGS: Amino Acid Composition

The application of Illumina sequencing to multiple fractions within the three rounds of biopanning allowed for a deeper insight into the processes involved in the biopanning rounds. Amplification-based and target-binding selection pressure could be quantified. Based on the results, we were able to sort sequences based on the underlying pressure. This helps in the identification of motifs, involved in the selective binding of arsenic.

Overall amino acid abundance is comparable to published data and the theoretical abundance of an NKK 12-mer library [28]. Whereas the deviating frequency on specific positions can be explained for some amino acids, amplification-induced changes (compare Figure 1 and Figure 2) are less described. Disulfide bridges are formed in the periplasm of *E. coli* via its *dsb* system. Presence of cysteine, especially in odd numbers, leads to covalent dimerization of PIII, preventing incorporation in and assembly of phage particles [29,30,31]. The display of cysteines in PVIII libraries is even more complicated, preventing protein processing and leader peptidase cleavage, resulting in cell death [32]. Therefore, cysteine, especially in odd numbers, is almost depleted in PIII M13 phage libraries. Proline at position 1 inhibits the signal peptidase, which cleaves the PIII leader sequence for the major protein, too [22,33,34]. Overabundance of prolines over most of the other positions has been described before by Malik et al., who found significantly reduced abundance of peptide inserts which tend to form α-helices [35].

Overabundance of alanine at +1 is also due to signal peptidase cleavage, as alanine is the only amino acid showing a significantly increased frequency at the first carboxy-terminal position after gram-negative bacterial signal peptidase cleavage [36,37].

The hydrophobic amino acids valine, phenylalanine, leucine, isoleucine, methionine and tryptophan are showing a decreased frequency after amplification. Although rarely discussed, it is assumed that N-terminal inserts hinder signal peptide cleavage of the preprotein, resulting in a polytopic membrane protein. Transport of the PIII preprotein to the cytosolic cell membrane, signal peptide cleavage and subsequent translocation of the protein through the membrane are required for synthesis of functional M13 phage [38,39]. Herman et al. found hydrophobic peptides, which could not be displayed with M13 phage but with T7 phage, most probably because the hydrophobic nature of the peptide inhibited correct phage assembly of M13 phage [40].

Amino acid composition after three rounds of biopanning shows no global frequency change for most amino acids, but position-dependent de-/increased frequencies. Cysteine shows an overall decreased abundance, as cysteine is sensitive to oxidation and the formation of intra- and inter-peptide disulfide bonds resulting in cysteine-containing sequences being involved in misfolded phage proteins. The aromatic amino acids tryptophan, phenylalanine, tyrosine and methionine show an overall decreased abundance, most probably as result of inhibition of phage assembly and missing signal peptide cleavage, when these amino acids are present in the insert. Exception are the tyrosine residues at +2 and +10, phenylalanine at +1 and methionine at +3, where they occur with increased abundance. Peptides, which still possess these amino acids after three rounds of biopanning are most likely subject to strong target-binding specific selection pressure, that counteracts the biased abundance described above.

Sequence logo calculation using pLogo [18] revealed formation of a consensus sequence after three rounds of biopanning in the elution fraction (FIMSVTKGRQVV) and in the stripping fraction (FHMPLTDPGQVQ) (only amino acids with highest binominal probability are shown, compare Figure 2). Both consensus sequences show phenylalanine at +1 and methionine at +3. Both amino acids possess an overall decreased abundance in these fractions. Furthermore, in the stripping fraction two carboxy-terminal glutamines at +10 and +12 are shown, whereas Rodi et al. reported glutamine to be less abundant at +10 and +12 in the naïve Ph.D.^TM^–12 library. This clearly indicates a directed selection pressure, which leads to enrichment of glutamines at these positions. The codons of both glutamines found in all motif-carrying sequences were TAG, which is the codon for the amber stop codon. In the suppressor strain, carrying *glnV*, which was used in this work, glutamine is inserted instead of the stop codon, preventing premature termination of the recombinant PIII.

### 4.3. NGS: Core Fraction Calculation

Core fractions were calculated in order to quantify and differentiate different selection pressures. The number of shared sequences within the fraction of the different biopanning rounds depends on the original number of unique sequences in the fractions and is limited by the minimum number of shared sequences (compare Table 2 and Table 3). Interestingly, the number of shared sequences relative to the minimum number of unique sequences in the included fractions is tenfold lower for the core fractions of elution and stripping compared to wash and input. This indicates an enrichment and thus loss of sequences on the fractions of the different biopanning rounds, suggesting a directed selection pressure. This observation clearly demonstrates that the experiments carried out in this work led to the enrichment of sequences and thus verifies the experimental setup.

When subtracting the different core fractions to calculate the difference sets, it becomes clear, that after subtracting the input and wash core fraction from the union ES, only few sequences are obtained. Subtraction of the input core fraction even leads to a reduction in sequences, which is not decreased anymore when the wash core fraction and the top 10% (and 25%) most occurring sequences were removed. The input core fraction was assumed to be mainly composed of sequences, that are subject to amplification-based selection pressure and possess a selection advantage due to fast propagation. Thus, this clearly indicates that the most abundant 10% (and 25%) percent of the naïve library possess a fast propagation-related growth advantage and are therefore included in the input core fraction. Furthermore, the wash core fraction is included in the input fraction, too. This shows that most sequences, which are removed by washing to get rid of low- and/or unspecific binding sequences, are also fast-propagating. However, calculating core fractions, unions of these fractions and subsequently subtracting the core sequence set of wash and input fractions, also leads to a severe depletion of sequences. Consequently, many probable candidate sequences that might possess good binding properties are removed, leading to a loss of information. Sequences that possess a good binding affinity to the target material and growth advantage due to fast propagation are removed from the library. Schönberger et al. and Rodi et al. identified target-binding sequences with high affinity, which were high abundant in the naïve library and after successful biopanning. Consequently, besides good binding affinity, these sequences possessed a growth-based selection advantage, too [14,26,41]. The sequence NYLPHQSSSPSR, which was identified by Schönberger et al. as a gallium binder, possessed arsenic-binding properties, too [42]. It was enriched in both elution and stripping fractions. However, due to its growth advantage, it was also found in all wash and stripping fractions and thus removed when the core fractions were calculated. It also contains the motif PSR, which was enriched in this study, too. This example illustrates, that the calculation of core sequences is a suitable method to exclude sequences with growth advantage and/or low or unspecific binding properties, however at the same time, the subtraction of sequences includes the risk of losing information and potential binding sequences.

The remaining peptide sequences, which were identified through calculation, are hidden deep in the fractions, as they only show low abundance. Yet, these sequences are enriched over the biopanning and would not have been identified with Sanger sequencing of single clones or Illumina sequencing of limited fractions at the end of three rounds of biopanning (compare Figure 3).

An alternative method for the removal of sequences, which are shared between two fractions, is suppression subtractive hybridization (SSH). This approach allows the comparison of DNA repertoires and the isolation of enriched sequences [43,44]. Vargas-Sanchez et al. could adapt this method to compare two phage display library populations, and were able to remove >96% of common non-specific sequences, shared between both fractions. The resulting population did possess an enriched affinity for the target [45]. However, the resulting population, although possessing enriched target affinity, is intrinsically disordered, complicating the identification of the best-binding sequences. When applied, we suggest implementing this physical DNA subtraction in the different biopanning rounds for target identification to further enrich target-specific sequence populations, but to omit SSH after the final round of enrichment. Thus, the phage display experiment benefits from the enhanced enrichment and, after completion, still presents the selection-based sequence abundance distribution.

### 4.4. NGS: Motif Enrichment

Motif enrichment analysis and motif comparison led on the one hand to the identification of motifs, which might mainly be part of fast-propagating sequences possessing growth advantage and likewise to the identification of motifs, which are part of potential target-binding sequences. The motifs PSR and LTD, e.g., have been described before, but specificity of sequences carrying this motif for the respective target material could not be shown [11]. With the data obtained from our study we assume that both motifs occur in sequences with growth advantage; however, for sequences carrying the motif QxQ, a potential target-specific selection pressure could be shown accompanying the growth advantage.

Hence, motif comparison allows the identification of sequences, possessing more than one motif involved in growth-related selection advantage. However, as discussed above, sequences can be both target-binding and fast-propagating. We recommend including further motif analysis into the existing phage display experiment databases, because knowledge about motif origin and assertiveness can help to distinguish identified sequences into target-specific and non-target related. Due to the strict core calculation conditions, many target-binding sequences may have been lost. Sequences that carry more than one enriched motif might be interesting candidates for further binding experiments, e.g., sequences QLQLDMDLSLHS and YQQQTSLHSPYA. Both sequences, however, do not possess the QxQ motif at the carboxy-terminal part of the sequence.

### 4.5. QxQ Motif: Metal- and Oxyanion Interaction

Metal binding of amino acids is often associated with histidine and cysteine, as most metal interactions are described for these two amino acids and their imidazole/thiolate group [46,47,48,49,50]. Most often, these interactions are supported by nearby peptide structures, cysteine interaction of complex molecules with metals has, however, been described even without surrounding structures [51]. Zinc, e.g., is coordinated predominantly by cysteine and histidine. Additionally, glutamic acid and aspartic acid have been described to interact via H-bonding [52].

The motifs discovered in this work, however, lack cysteine (except CHMPLTDPGQVQ) and possess histidine at +2 in QxQ containing motifs and in the SxHS motif. Glutamic acid is not present in the sequences. Aspartic acid is found at +7 in QxQ motif family sequences. Methionine, the second sulfur-containing amino acid, has been found in the sequences at +3 and has been described as metal-binding earlier [53,54].

Special emphasis here is put on the question of if the conserved motif QxQ is involved in the binding of arsenic and a result of the target-specific selection pressure. QxQ motifs have been described to be involved in interaction with Ni^2+^ and other alkali metal ions [55,56,57]. The broader metal binding ability of glutamine was further described for copper [58], whereas Chiera et al. could even show a major impact of glutamine residues and its H-bonds on the stability of copper-binding peptides [59]. Glutamine was also described to be involved in the surface binding and structure forming of platinum-binding peptides [60] Furthermore, it is described as being abundant in the coordination spheres of metal ions [61,62]. Mitsui et al. discovered the QxQ motif in zinc fingers [63].

We propose that the carboxy-terminal QxQ motif is involved in the interaction with the oxyanion (occurring mostly as As(V) H_2_AsO_4_^−^/HAsO_4_^2−^ [25]). The interaction of proteins and arsenic oxyanions occurs mainly over the thiolate group of cysteine [64]; however, hydrogen bonds are involved [65], which can be formed by glutamine, too [66].

Therefore, the interaction is most probably based on the formation of hydrogen bonds, as the main interactions of glutamine are H-bond based [67]. The functional group of the side chain of glutamine is a carboxamide, allowing interaction over the carbonyl- or the secondary amine group. Both have been described to be involved in metal coordination [68,69,70]. In addition, cysteine, serine and asparagine have been described as ligands for the interaction with the oxyanions of molybdenum and arsenic [71]. An explanation for the occurrence of the QxQ motif in many of the identified sequences could be that glutamine replaces asparagine in the formation of H-bonds, which has also been described to form H-bonds with arsenite [72].

### 4.6. QxQ Motif: Biological Occurrence

Interaction of oxyanions with proteins is performed by coordinating the oxyanion in an oxyanion hole. Ménard et al. found glutamine to be involved in the stabilization and structure formation of the oxyanion hole of papain [73]. The toxicity of arsenic and the oxyanions of, e.g., vanadium and molybdenum are caused by its ability to mimic phosphate. Ubiquitous phosphate-utilizing enzymes and pathways are either inhibited by arsenic or used for its metabolization, i.e., arsenic is taken up by the phosphate transport system [74,75,76].

Using PROSITE [77,78,79] we discovered, that the consensus sequence QxQ is part of many serine/threonine kinases (EC 2.7.11.-), tyrosine kinases (EC 2.7.10.-), polyphosphate kinases (2.7.4.-) and other enzymes, transferring phosphorus-containing groups (2.7.-.-). We therefore hypothesize, that the QxQ motif is involved in the interaction with the oxyanion of arsenic and can interact with phosphate, too. This corresponds to publications, that did identify QxQ motifs in tyrosine kinases [80] and phosphatases [81]. Shi et al. found QxQ motifs in close proximity to the phosphate binding site especially in PPM protein phosphatases [81].

Silver et al. mapped several genes and enzymes, which are involved in the bacterial oxidation and reduction of arsenic. QxQ and QxxQ motifs can be found in various proteins of different bacteria responsible for oxyanion transport, binding and oxidation/reduction reactions of arsenic [82]. Furthermore, for the bacterial arsenate reductase ArsR from *E. coli,* binding of As^III^ to the cysteines Cys32, Cys34 and Cys37 is described, whereas a QxQ motif is found on position 42–44. [3]. ArsC binds the arsenic oxygen through initial non-covalent binding and subsequent interaction with only one cysteine, which could be provided by CHMPLTDPGQVQ [83]. These findings indicate the involvement of QxQ motif-carrying sequences in the interaction with arsenic.

## 5. Conclusions

Three rounds of biopanning against on-column immobilized arsenic on a cationic ion exchange material were performed. All major, phage-containing fractions and the naïve phage library have been Illumina sequenced to compare the data with the traditional phage display experimental setup and Sanger sequencing of the elution and stripping fraction after the third biopanning round. Sanger sequencing revealed two high abundant sequences (11/68). Comparison with NGS data showed that sequences obtained through Sanger sequencing only cover sequences, which were highly abundant in the naïve library, too. However, sequence variations and phage display-typical selection biases remained unknown after Sanger sequencing. Usage of bioinformatics tools and core fraction calculation revealed the highly enriched motifs QxQ and SxHS in different sequences. Further motif analysis led to the identification and classification of derived motifs into growth-related and target-specific selection pressure enriched sequences. Comparison with published proteins and functions supported the potential arsenic-binding function of the QxQ motif leading to the discovery of diverse candidate peptide sequences, which may be able to bind to arsenic.

The aim of this study was to compare and verify traditional phage display setup using Sanger sequencing of limited number of singles clones with a larger number of sequences, derived with Illumina and to identify phage display artefact interfering with the successful identification of target-binding sequences. We could show that Illumina sequencing greatly increases the insight into the phage display selection mechanisms. For future phage display experiments, the authors recommend the extensive usage of bioinformatics tools and databases like PuLSE, pLogo, MEME, ProSite, Phastpep, PepSimili, PhD7Faster, Sarutop and, e.g., MimoDB (incomplete list of suitable reviews and literature [84,85,86,87,88,89,90]). These tools and databases help to unravel NGS results, discover enriched motifs, separate fast-propagation biased sequences and other target-unrelated peptides and to rank identified peptides for their occurrence. Special consideration should be given to the database www.48hd.cloud [23], a database and company by Derda et al. for the evaluation of NGS data of phage display experiments, which is still under construction. Our study may also serve as a guideline for future phage display experiments and evaluations. Next generation sequencing allowed a detailed description of the sequence variations over the three rounds of biopanning and, by comparison of different fractions, we could not only show which motifs are enriched and hidden below high-abundant sequences, but also discriminate growth-bias related sequences. Furthermore, changes in the proportion of amino acids were assigned to the respective selection pressure, as was shown, e.g., for the amplification-biased reduction of hydrophobic amino acids. We suggest Illumina sequencing of various fractions in future phage display experiments and additionally to the usage of bioinformatics tools, we strongly recommend the internal comparison of the obtained fractions to gain a better understanding of the occurrence of finally enriched motifs.

However, in this study, a single molecule was chosen as a target for phage display. The expected repertoire of identified ligands is therefore very specific. More complex targets might result in larger and more diverse ligand species and therefore complicate the underlying calculations. Furthermore, the sequencing, calculation and evaluation of sequencing data is always limited to in silico presentation of results. Only peptide synthesis, alanine scanning, site-directed mutagenesis and peptide binding experiments can finally verify these data. The in-depth insight into the underlying mechanisms of the phage display experiments that were carried out allows a better preselection for subsequent in situ binding studies. Most promising candidate sequences of this study will be subject to further binding studies, as well.

## Figures and Tables

**Table 1:** Peptide sequences, pI and occurrence of the combinatorial Ph.D.^TM^–12 phage library LOT 0151606 (New England Biolabs Inc., Ipswich, MA, USA) identified with Sanger sequencing after three rounds of biopanning against arsenic oxyanions immobilized on quaternary amines.

Peptide Sequence	pI	Occurrence	Peptide Sequence	pI	Occurrence
FHMPLTDPGQVQ	5.08	11/68			
SIHSVTKGRYPV	9.99	11/68			
MKAHHSQLYPRH	9.99	2/68			
ANGSEYNLLQQS	4.00	1/68	DFPRTKSETRAP	8.75	1/68
DGMTKPAQHTNR	8.75	1/68	DPMQKSHLVSQS	6.74	1/68
DVLQPEGLTIPL	3.67	1/68	EDSGLASEKIAR	4.68	1/68
ERNVTSDDPGSI	4.03	1/68	FSDRVGSILNSP	5.84	1/68
GAISDYTPSQFY	3.80	1/68	GSAARTISPSLL	9.75	1/68
GVAAAVSVSNAS	5.52	1/68	GYLGSYRAHEDS	5.32	1/68
HSPALDRLHGIP	6.92	1/68	LPITEKEPYDKF	4.68	1/68
LQTYDNPAKSIN	5.83	1/68	NEVNNSSGAPKQ	6.00	1/68
NLTYKQINPAAF	8.59	1/68	NNHNGPDVTYWV	5.08	1/68
NYLPHQSSSPSR	8.75	1/68	QARTAMSLEQHL	6.75	1/68
QCLASCLGPQRV	8.07	1/68	RISYKPDSWQAS	8.59	1/68
RLPSYTTGLIAN	8.75	1/68	SMSSGLTSNKSY	8.31	1/68
SDNLHYTLLPMH	5.92	1/68	SNKNLDTRILTK	9.99	1/68
SHMLSSEWESAS	4.51	1/68	STNLYNTVAYQD	3.80	1/68
SITELLNAAHST	5.22	1/68	SYMWATGSPLAY	5.24	1/68
SLSPAGYTRLSL	8.46	1/68	TGKLIESSPDSI	4.37	1/68
THSEPYYPHSHK	6.74	1/68	TIKEPFPNRDLY	5.73	1/68
TISAFTSFMPTN	5.19	1/68	VRPTTEYMETSM	4.53	1/68
WGVTKPIRTSTL	11.00	1/68	QINQDSLHTPAA	5.08	1/68
YDAIQRPTGQLS	5.84	1/68	YQRPANLSMEDR	6.07	1/68

**Table 2:** Read and unique sequence occurrence acquired by Illumina sequencing in the different fractions of the three biopanning (BP) rounds, in the naïve library Ph.D.^TM^–12 LOT 0151606 and its amplification.

**Biopanning**	**Fraction**	**Reads**	**Unique Sequences**
	naïve library Ph.D.^TM^–12 LOT 0151606	133,163	97,563
	amplification of naïve library	85,533	59,375
biopanning 1 BP1	input	87,883	67,705
	wash	5271	2915
	elution	3975	2563
	stripping	124,565	16,235
biopanning 2 BP2	input	109,784	82,399
	wash	3274	1185
	elution	72,950	3536
	stripping	20,167	1999
biopanning 3 BP3	input	74,389	20,331
	wash	2001	999
	elution	2373	1268
	stripping	2828	1487

**Table 3:** Summary of read and unique sequence quantities of core fractions (sets) calculated in this work for a phage display experiment with three rounds of biopanning against on-column immobilized arsenic.

Core fFaction (Set)	Read Number	Unique Sequences
∩I	15,027	2912
∩W	4931	381
∩E	209	56
∩S	5304	74
ES	1753	113
ES\W	613	48
ES\I	51	14
ES–I–W	51	14
$\mathrm{ES}–I–W\backslash \mathrm{na}ï.\mathrm{lib}._{\mathrm{TOP}10\%}$	51	14
$\mathrm{ES}–I–W\backslash \mathrm{na}ï.\mathrm{lib}._{\mathrm{TOP}25\%}$	41	13

**Table 4:** Occurrence of the three most abundant sequences FHMPLTDPGQVQ, SIHSVTKGRYPV and MKAHHSQLYPRH of Sanger sequencing(^+^) in comparison to the Illumina sequencing results (1–5) of selected fractions of three rounds of biopanning against on-column immobilized arsenic using the Ph.D.^TM^–12 phage library from New England Biolabs. Occurrence of the sequences is given relative to the overall read number and in percentage. Selected fractions are: (1) naïve library, (2) amplification of naïve library, (3) input into the three rounds of biopanning, (4) elution fraction of third biopanning round, (5) stripping fraction of third biopanning round.

Fraction	FHMPLTDPGQVQ		SIHSVTKGRYPV		MKAHHSQLYPRH	
0 Sanger seq.^+^	11/68	(16.18%)	11/68	(16.18%)	02/68	(2.94%)
1 naïve library	143/143,424	(0.10%)	110/143,424	(0.08%)	232/143,424	(0.16%)
2 ampli. naï. lib.	115/85,533	(0.13%)	99/85,533	(0.12%)	208/85,533	(0.24%)
3 input BP1	84/87,883	(0.10%)	69/87,883	(0.08%)	160/87,883	(0.18%)
4 elution BP3	134/2373	(5.65%)	147/2373	(6.19%)	28/2373	(1.18%)
5 stripping BP3	252/2828	(8.91%)	164/2828	(5.80%)	41/2828	(1.45%)

**Figure A2:**
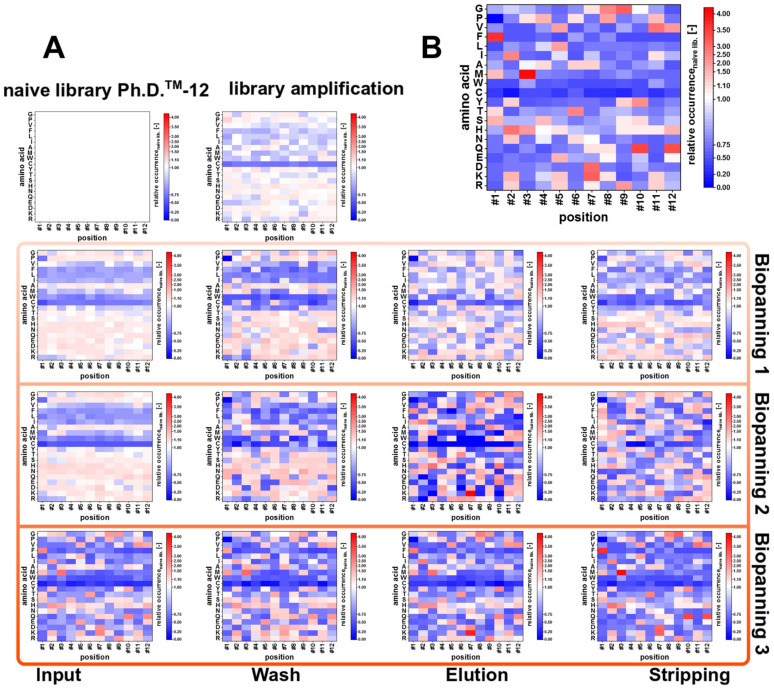
Amino acid composition of the fractions of three rounds of biopanning against on-column immobilized arsenic (**A**). Shown is the relative occurrence of each amino acid at each position of the randomized 12-mer sequence displayed on the outmost part of M13KE phage in the Ph.D.^TM^–12 phage library (New England Biolabs, Ipswich, MA, USA) relative to the percentage of occurrence of the amino acids in the naïve library. (**B**) shows an enlarged view of the stripping fraction of biopanning round 3.

## Appendix A

**Table A1:** Amino acid frequency (%) of the naïve Ph.D.^TM^–12 phage library LOT 0151606 (New England Biolabs, Ipswich, MA, USA) after Illumina sequencing of 133,163 reads, resulting in 97,563 unique sequences.

Amino Acid		Frequency in Naïve Library (%)
arginine	R	5.09
lysine	K	2.39
aspartic acid	D	4.00
glutamic acid	E	2.60
glutamine	Q	4.14
asparagine	N	4.65
histidine	H	5.40
serine	S	10.80
threonine	T	9.49
tyrosine	Y	3.80
cysteine	C	1.00
tryptophan	W	1.97
methionine	M	3.07
alanine	A	6.88
isoleucine	I	3.44
leucine	L	9.13
phenylalanine	F	2.92
valine	V	4.97
proline	P	10.06
glycine	G	4.21
